# Existing Models of Maternal Death Surveillance Systems: Protocol for a Scoping Review

**DOI:** 10.2196/resprot.5758

**Published:** 2016-10-11

**Authors:** Saloua Abouchadi, ASM Shahabuddin, Wei Hong Zhang, Tabassum Firoz, Yvon Englert, Chakib Nejjari, Vincent De Brouwere

**Affiliations:** ^1^ Ecole Nationale de Santé Publique Rabat Morocco; ^2^ Institute of Tropical Medicine Maternal and Reproductive Health Unit Antwerp Belgium; ^3^ School of Public Health Université Libre de Bruxelles Brussels Belgium; ^4^ The Warren Alpert Medical School Department of Medicine Brown University Providence, RI United States; ^5^ Hôpital Erasme Laboratoire de Recherche en Reproduction Humaine Université Libre de Bruxelles Brussels Belgium

**Keywords:** maternal mortality, surveillance systems, completeness, usefulness, scoping review, protocol

## Abstract

**Background:**

Maternal mortality measurement remains a critical challenge, particularly in low and middle income countries (LMICs) where little or no data are available and maternal mortality and morbidity are often the highest in the world. Despite the progress made in data collection, underreporting and translating the results into action are two major challenges that maternal death surveillance systems (MDSSs) face in LMICs.

**Objective:**

This paper presents a protocol for a scoping review aimed at synthesizing the existing models of MDSSs and factors that influence their completeness and usefulness.

**Methods:**

The methodology for scoping reviews from the Joanna Briggs Institute was used as a guide for developing this protocol. A comprehensive literature search will be conducted across relevant electronic databases. We will include all articles that describe MDSSs or assess their completeness or usefulness. At least two reviewers will independently screen all articles, and discrepancies will be resolved through discussion. The same process will be used to extract data from studies fulfilling the eligibility criteria. Data analysis will involve quantitative and qualitative methods.

**Results:**

Currently, the abstracts screening is under way and the first results are expected to be publicly available by mid-2017. The synthesis of the reviewed materials will be presented in tabular form completed by a narrative description. The results will be classified in main conceptual categories that will be obtained during the results extraction.

**Conclusions:**

We anticipate that the results will provide a broad overview of MDSSs and describe factors related to their completeness and usefulness. The results will allow us to identify research gaps concerning the barriers and facilitating factors facing MDSSs. Results will be disseminated through publication in a peer-reviewed journal and conferences as well as domestic and international agencies in charge of implementing MDSS.

## Introduction

In September 2011, the World Health Organization Commission on Information and Accountability for Women's and Children's Health announced 10 recommendations which focused on strengthening country and global accountability [1]. To achieve better results countries need to improve their health information systems, develop civil registration and vital statistics systems, implement innovative approaches to count and review maternal deaths, and monitor progress [2].

Maternal mortality measurement remains a challenge especially in low and middle income countries (LMICs) [2]. Only five among those 139 countries have functional civil registration and vital statistics systems, which are the preferred source of data for counting deaths and defining their causes [3]. In the absence of such systems, censuses, household surveys, and special studies are currently used to collect retrospective data on maternal mortality. Consequently, the maternal mortality ratio is not often accurate. In addition, the uncertainty of statistics derived using these methods tends to be very large, and data are not generally available at the subnational level. Such limits make data inappropriate for proactive response, planning, or resource allocation [4].

On the path of ending preventable maternal mortality, the Maternal Death Surveillance and Response approach was launched in 2012 by the World Health Organization and partners after the failure of many LMICs to implement the approach Beyond the Numbers, which had been launched in 2004 [5].

Despite the progress made in collecting data, many questions remain unanswered such as how to better implement the various maternal death surveillance systems (MDSSs). The underreporting and poor use of results of MDSSs for action are two major additional challenges in LMICs. At present, there are no systematic or scoping reviews published that address the question about MDSS performance and the use of their results for decision making.

Considering the importance of the issue, we propose a protocol for a scoping review covering the existing models of MDSSs with the objective of better understanding the factors that influence the completeness and usefulness of MDSSs.

We propose to map and synthesize the available literature to identify and describe current models of MDSSs and explore factors affecting their completeness and usefulness.

## Methods

### Protocol Design

We plan to undertake a scoping review, an approach which has been growing in popularity for the last few years as a useful tool that can provide a broad overview of a topic [6]. The scoping review methodology was chosen for this particular study because there is little literature in this field. In addition, a scoping review will facilitate the identification of knowledge gaps and opportunities that exist pertaining to an emerging subject of interest [6].

Our protocol was developed by using the York methodological framework proposed by Arksey and O’Malley [7], detailed by Levac et al [8], and further refined by the Joanna Briggs Institute (JBI) [9,10]. This methodology outlines a 5-stage approach: (1) identify the research question; (2) identify relevant studies; (3) select studies; (4) chart the data; and (5) collate, summarize, and report the results, with an optional consultation exercise. The first author of this paper developed the draft protocol which was revised upon receiving feedback from all coauthors. Consideration will be given to revising the methodology as needed throughout the review process.

### Inclusion Criteria

The inclusion criteria consist of three parts as identified by the JBI: participants, concept, and context [9].

#### Participants

This scoping review will include women of reproductive age deceased during pregnancy, childbirth, or puerperium until one year after termination of pregnancy. *Women of reproductive age* refers in general to all women aged 15 to 49 years [11]. According to the International Classification of Diseases, Tenth Revision, a maternal death is a death of a woman while pregnant or within 42 days of termination of pregnancy [12]. These deaths are subdivided into two groups: (1) maternal death due to direct cause, indirect cause, or unknown/unspecified cause and (2) other deaths during pregnancy, childbirth, and puerperium due to coincidental causes [12]. A late maternal death is the death of a woman from direct or indirect causes more than 42 days but less than one year after termination of pregnancy [12].

#### Concept: Intervention and Outcomes

We will include all the reporting systems related to the maternal mortality surveillance that detect and/or monitor maternal deaths, help understand the underlying factors contributing to the deaths, and stimulate and guide actions to prevent future deaths. MDSSs include review systems such as audits, maternal death reviews, and confidential enquiries. For describing the MDSS implementation process and challenges, we will consider studies performed both at national and subnational level.

We will focus on two specific attributes of a surveillance system: external completeness and usefulness [13]. External completeness applies to the reporting process and relates to whether the data available to the surveillance system reflect the true number of cases affected by a given condition. The numbers of maternal deaths reported will be compared to the estimated number when the information is available. Usefulness implies that surveillance results are used for public health action [14,15]. This attribute will be considered according to the objectives of the MDSS. We will describe the MDSS effect on decision making at national and subnational level and identify actions that have been taken as a result of MDSS outputs.

#### Context

We will not limit the context of our scoping review to a particular setting or country.

### Types of Studies

For the purpose of our scoping review, we will include both quantitative and qualitative research studies. Other publications such as opinion papers, reports, and government guidance will be also taken into consideration.

### Information Sources and Search Strategy

The search strategy will include published and gray literature. As recommended by the JBI, a three-step search strategy will be utilized [9]. The first step is an initial limited search of two online databases which are relevant to our topic: PubMed and Web of Science. Medical subject headings terms from PubMed have been used at the start to determine the words used to search in PubMed. The search strategy can be found in Multimedia Appendix 1. We have combined search terms focused on maternal mortality, surveillance systems, and attributes of surveillance systems (completeness and/or usefulness).

This initial search will be then followed by an analysis of the text words contained in the title and abstract of retrieved papers and of the index terms used to describe the articles. A second search using all the identified keywords and the index terms specific to each database will be undertaken across all accessible databases and websites. The search will then be performed using the following additional electronic databases: POPLINE, Cochrane Effective Practice and Organisation of Care Group, and Public Health Interventions Cost Effectiveness Database. We will conduct further searches in the following sources of gray literature: WHO Library, *Banque de données en santé publique*, African Journals OnLine, Maternal Death Surveillance and Response Action Network, INDEPTH Network, and Google Scholar.

The search strategy will be modified as necessary to accommodate database differences. Additional keywords, sources, and potentially useful search terms may be discovered and incorporated into the search strategy. Finally, the reference lists of all identified reports and articles will be searched for additional studies. Search results will be imported into reference management software (Reference Manager, Thomson Reuters Corp), and duplicate citations will be removed. No restrictions of language or date limit will be applied for our search strategy.

### Study Selection Process

Two reviewers (SA and ASh) will independently screen titles and abstracts to check for relevance to the review. The reviewers will exchange at the middle and the end of screening process to discuss their selection of articles and to refine the search strategy, if needed. Additional keywords, sources, and potentially useful search terms may be discovered and incorporated into the search strategy.

Using the same process, the reviewers will subsequently screen the full text and apply the inclusion criteria for potentially relevant articles that were not excluded by looking at the title or the abstract. All discrepancies between reviewers will be resolved by a single arbitrator (VDB).

### Extraction of the Results

A draft tool has been developed according to the JBI results extraction instrument to record the information from the articles [9]. The extracted data will include study characteristics (eg, study population, setting, study time period, data sources, study size, study design). For describing the MDSS, extracted data will be based on key elements for the description of a surveillance system [13,14,16] which include national legislation, main stakeholders, surveillance objectives, type of surveillance, geographic coverage, time of data collection, data sources/data providers, reporting process/data flow, case definition, type of data collected, data management, resources needed, data analysis and dissemination of results, participant privacy/ data confidentiality/system security, and eventually healthcare system constraints. To evaluate the MDSS, two attributes will be considered: completeness and usefulness (described in Multimedia Appendix 2). The two reviewers will independently extract data from three articles to ensure interreader reproducibility. The form will be then sent to all team members for final comments and suggestions before implementing it for screening articles. Data extraction will be an iterative process; the charting table may be updated if other additional unforeseen data are identified.

## Results

**Figure 1:**
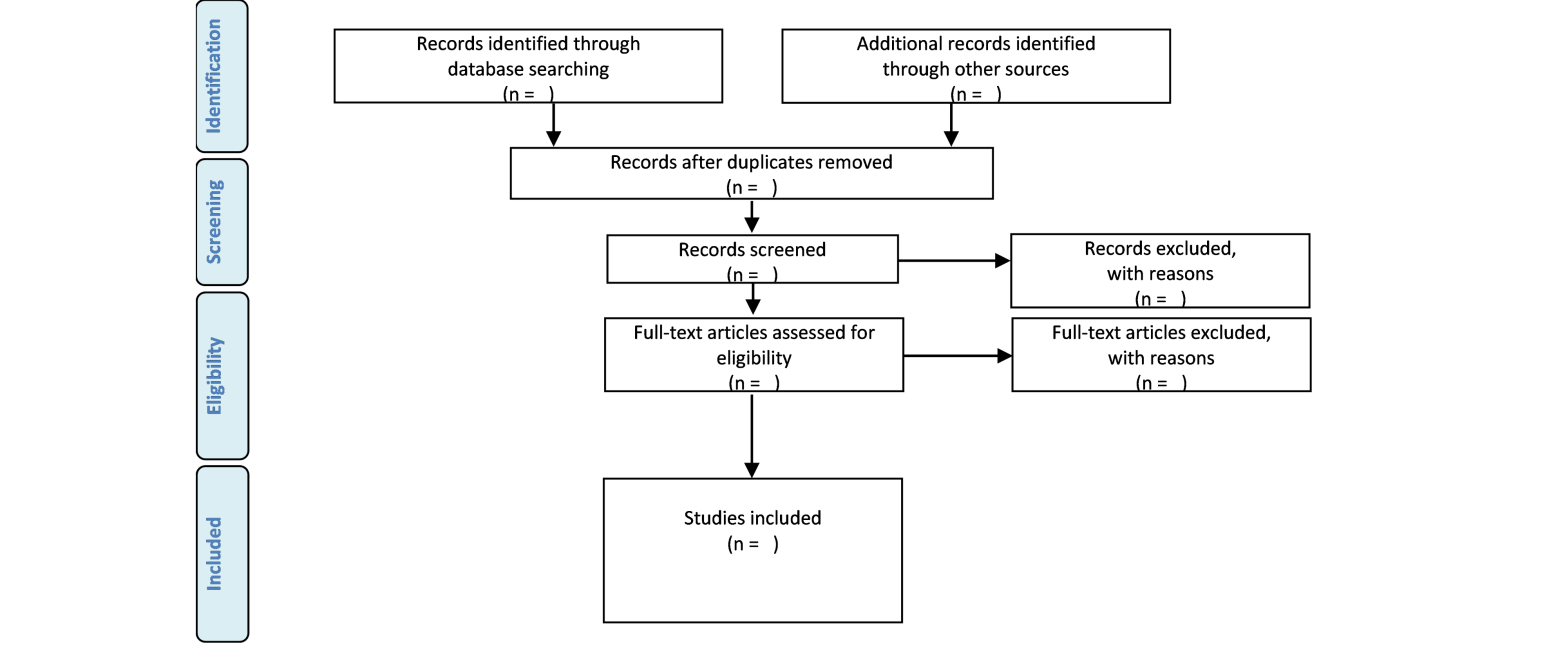
Preferred Reporting Items for Systematic Reviews and Meta-Analyses (PRISMA) flow diagram for the scoping review process [17].

Currently, the abstracts screening is under way and the first results are expected to be submitted for publication by mid-2017. The review decision process and its results will be detailed using the “Preferred Reporting Items for Systematic Reviews and Meta-Analyses (PRISMA) flow diagram (Figure 1) [17]. Data analysis will involve quantitative analysis (eg, frequency analysis) of existing models of MDSSs.

The synthesis will also include qualitative analysis using a thematic analysis [18] of the factors that influence completeness and usefulness. Categories will be generated using the main themes and text will be coded manually according to each category. Broad categories of themes can be grouped as follows [13]:

1. Factors related to the health care system (eg, lack of personnel)

2. Factors related to the data providers (eg, lack of interest, training, adherence, confidentiality issues/concerns of the data providers, proper supervision, knowledge on the objectives, usefulness of the surveillance system)

3. Factors related to the structure and functionality of the system (eg, notification process, reporting form, electronic data entry system, surveillance protocol, resources, visibility of the surveillance system and its data)

4. Factors related to external circumstances or constraints (eg, government ownership and commitment, liability and punitive measures).

Additional steps include annotating emerging themes and patterns and readjusting the categories and relationships between them, testing emergent propositions through systematic searches of coded text, and searching for alternative explanations through systematic searches of uncoded text.

The synthesis of the reviewed materials will be presented in a tabular form complemented by a narrative format. The tables will show the results as in the chart for results extraction in Multimedia Appendix 2. The narrative summary, made by categories, will describe how the results relate to the review objectives. The results will be classified in main conceptual categories that will be obtained during the results extraction.

Specific factors that influence completeness and usefulness will be grouped by domain, and the final list of factors will be determined and agreed on by all the authors.

## Discussion

This scoping review will provide a broad overview and comparison of different MDSSs. We will describe factors related to their completeness and usefulness. Currently, 103 LMICs are in the process of implementing MDSSs among which 46% apparently are functioning [5]. However, informal discussions with several people in charge of implementing MDSSs show that the systems are not functioning well, and no paper showing any type of empirical result has been published so far. Furthermore, the barriers to completeness and translating the recommendations generated by MDSSs into action have yet to be examined in depth.

By including all MDSSs, we will capture findings from those well-resourced settings. The lessons learned from successful experiences such as the implementation of Confidential Enquiry into Maternal Deaths (United Kingdom) and in the surveillance of morbidity and near miss case reviews may contribute to further improving and enhancing MDSSs in LMICs.

Potential gaps in the field will be identified and the results will inform future research directions on barriers and facilitating factors of MDSSs; hence, we expect our findings will be useful for the country teams and United Nations agencies in charge of implementing of MDSSs and interesting to networks and researchers who are working on this topic. The results will also be published in a peer-reviewed journal.

We will use rigorous and transparent methodology by following the JBI guidelines for scoping reviews. To ensure a broad literature search, the search strategy includes five electronic bibliographic databases, reference lists of items, and the most important sources of gray literature. However, there is a possibility of missing potentially relevant articles due to noninclusion of other sources.

The data-charting form will be pretested by the reviewers and revised as necessary before implementation. Each citation and article will be reviewed by two independent reviewers. Our use of a bibliographic manager will ensure that all citations and articles will be properly accounted for in the process.

Finally, it may not be possible to develop recommendations for practice from the results of this scoping review as no assessment of methodological quality or rating of level of evidence will be carried out.

## Appendix

Multimedia Appendix 1 Search term strategy in PubMed (July 2016).

Multimedia Appendix 2 Draft of the data-charting form.

## Abbreviations

JBI: Joanna Briggs Institute
LMICs: Low and middle income countries
MDSS: Maternal Death Surveillance System
PRISMA: Preferred Reporting Items for Systematic Reviews and Meta-Analyses
