# Supplementary material for: Existing Models of Maternal Death Surveillance Systems: Protocol for a Scoping Review
Source: JMIR Res Protoc. 2016 Oct 11;5(4):e197. doi: 10.2196/resprot.5758 (PMC5080526; doi:10.2196/resprot.5758)
Supplement: Multimedia Appendix 1 [file resprot_v5i4e197_app1.pdf]

Appendix1. Search strategy for Pubmed <1946 to Present>

Search	Query
**Participants**	
#1	"Maternal Mortality"[Mesh]
#2	"Maternal Death"[Mesh]
#3	"Mothers/mortality"[Mesh]
#4	"Pregnancy/mortality"[Mesh]
#5	Maternal mortalit*
#6	Maternal death*
#7	Pregnan* mortalit*
#8	Pregnan* death*
#9	OR/#1 - #8
**Intervention of interest**	
#10	"Population Surveillance"[Mesh]
#11	"Public Health Surveillance"[Mesh]
#12	"Mandatory Reporting"[Mesh]
#13	"Registries"[Mesh]
#14	"Clinical Audit"[Mesh]
#15	"Medical Audit"[Mesh]
#16	"Clinical Governance"[Mesh]
#17	Surveillance*
#18	Report*
#19	Monitor*
#20	Audit*
#21	Review*
#22	Confidential enquir*
#23	OR/#10 - #22
**Outcomes**	
#24	Complet*
#25	Use*
#26	Underreport*
#27	Under report*
#28	Misreport*
#29	Unreport*
#30	Misclassificat*
#31	Hidd*
#32	OR/#24 - #31
**Context**	not limit to a particular setting or country
#33	#9 AND #23 AND #32

Search terms expanded.
