# Supplementary material for: Existing Models of Maternal Death Surveillance Systems: Protocol for a Scoping Review
Source: JMIR Res Protoc. 2016 Oct 11;5(4):e197. doi: 10.2196/resprot.5758 (PMC5080526; doi:10.2196/resprot.5758)
Supplement: Multimedia Appendix 2 [file resprot_v5i4e197_app2.pdf]

Appendix2. Draft of the data-charting form

**Study details and characteristics extraction**
a. Authorship
b. Source
c. Year of publication
d. Setting / Country
e. Size of target population
f. Aims/purpose
**Results extraction**
a. <u>Description of the MDSS</u>
National legislation
Main stakeholders
Surveillance objectives
Type of surveillance<br>– Passive vs. active<br>– Compulsory vs. voluntary<br>– Comprehensive vs. sentinel
Geographic coverage (regional, national)
Time of data collection
Data sources/data providers
Reporting process, data flow<br>– Levels of reporting system<br>– Type of reporting (Paper-based / Electronic)
Case definition of maternal death
Type of data collected (Specification of the information to be reported)<br>– Case-based and aggregated<br>– Variable specification<br>– Frequency of data collection
Data management<br>–Data entry: Web-based, Interface-mediated data entry, Open-source software<br>–Database architecture: Centralised / Server selection<br>–Editing , Storage, Back up, Transfer of data
Resources needed<br>–Types<br>–Amount<br>–Timing
Data analysis and dissemination of results
Participant privacy, data confidentiality, and system security
Healthcare system constrains
b. <u>Quality attributes</u>
Completeness<br>–Measure<br>–Evaluation method<br>–Factors of completeness
Usefulness<br>Inventory of actions that have been taken in conjunction with the surveillance system
